# Thyroid-Stimulating Hormone Favors Runx2-Mediated Matrix Mineralization in HOS and SaOS2 Cells: An In Vitro and In Silico Approach

**DOI:** 10.3390/molecules27030613

**Published:** 2022-01-18

**Authors:** Ramajayam Govindan, Mohamed El-Sherbiny, Khalid Mohamed Morsy Ibraheem, Srinivasan Narasimhan, Mohamed EL-Dosoky Mohamed Salama, Fazil Ahmad, Selvaraj Jayaraman, Vishnu Priya Veeraraghavan, Srinivasan Vengadassalapathy, Surapaneni Krishna Mohan, Vidhya Rekha Umapathy, Gayathri Rengasamy, Shazia Fathima Jaffer Hussain, Maheshkumar Poomarimuthu, Senthilkumar Kalimuthu

**Affiliations:** 1 Multidisciplinary Research Unit, Madurai Medical College, Madurai 625020, India; makeshkumar87@gmail.com; 2 Department of Endocrinology, Dr.A.L.M. Post Graduate Institute of Basic Medical Sciences, University of Madras, Chennai 600113, India; n.srini2000@unom.ac.in; 3 MDepartment of Basic Medical Sciences, College of Medicine, Almaarefa University, Riyadh 71666, Saudi Arabia; msharbini@mcst.edu.sa; 4 Department of Anesthesia Technology, College of Applied Medical Science in Jubail, Imam Abdulrahman Bin Faisal University, Jubail 35816, Saudi Arabia; kmibraheem@iau.edu.sa (K.M.M.I.); fmahmad@iau.edu.sa (F.A.); 5 Department of Neuroscience Technology, College of Applied Medical Science in Jubail, Imam Abdulrahman Bin Faisal University, Jubail 34221, Saudi Arabia; mesalama@iau.edu.sa; 6 Centre of Molecular Medicine and Diagnostics (COMManD), Department of Biochemistry, Saveetha Dental College & Hospital, Saveetha Institute of Medical & Technical Sciences, Chennai 600077, India; vishnupriya@saveetha.com (V.P.V.); gayathri.sdc@saveetha.com (G.R.); 7 Department of Pharmacology, Saveetha Institute of Medical & Technical Sciences, Saveetha Medical College and Hospital, Chennai 602105, India; srinivasanv.smc@saveetha.com; 8 Departments of Biochemistry, Molecular Virology, Research, Clinical Skills & Simulation, Panimalar Medical College Hospital & Research Institute, Varadharajapuram, Poonamallee, Chennai 600123, India; krishnamohan.surapaneni@gmail.com; 9 Department of Public Health Dentistry, Sree Balaji Dental College and Hospital, Pallikaranai, Chennai 600100, India; drvidhyarekha@gmail.com; 10 Department of Oral and Maxillofacial Pathology, Ragas Dental College and Hospitals, Chennai 600119, India; shaziafathimarizwan@gmail.com; 11 Central Research Laboratory, Melmaruvathur Adhiparasakthi Institute of Medical Sciences and Research, Melmaruvathur 603319, India; senthilbhus@gmail.com

**Keywords:** TSH, Runx2, ALP, SaOS2, CREB, ELK1

## Abstract

Osteoporosis is a skeletal disease that is both systemic and silent characterized by an unbalanced activity of bone remodeling leading to bone loss. Rising evidences demonstrate that thyroid stimulating hormone (TSH) has an important role in the regulation on the metabolism of bone. However, TSH regulation on human osteoblast essential transcriptional factors has not been identified. Current study examined the role of TSH on human osteoblastic Runx2 expression and their functional genes by in vitro and in slico analysis. Human osteoblast like (HOS and SaoS-2) cells were cultured with DMEM and treated with hTSH at the concentration of 0.01 ng/mL and 10 ng/mL. After treatment, osteoblastic Runx2 and IGF-1R beta expression were studied using RT-PCR and western blot analysis. TSH treatment induced osteoblastic essential transcriptional factor, Runx2 in HOS and SaOS2 cells on 48 h duration and elevated the expression of IGF-IR β gene and Protein in SaoS-2 cells. TSH also promotes Runx2 responsive genes such as ALP, Collagen and osteocalcin in SaOS2 cells on day 2 to day 14 of 10 ng/mL of treatment and favors’ matrix mineralization matrix in these cells. In addition, TSH facilitated human osteoblastic cells to mineralize their matrix confirmed by day 21 of alizarin red calcium staining. In silico study was performed to check CREB and ELK1 interaction with Runx2. Results of in silico analysis showed that TSH mediated signalling molecules such as CREB and ELK1 showed interaction with Runx2 which involve in osteobalstic gene expression and differentiation. Present findings confirm that TSH promotes Runx2 expression, osteoblastic responsive genes and bone matrix formation.

## 1. Introduction

Thyroid stimulating hormone (TSH) is known to regulate bone metabolism and their biologically active receptors present in the cells of bone. TSH can directly act on the skeleton [1,2,3,4]. Stimulation of the TSH receptor (TSHR) in osteoclast, suppresses the resorption of bone [1]. In a rodent model, administration of TSH intermittently stimulates osteoblastic bone formation and prevents ovariectomy-induced bone loss [2,3,4]. Studies also found that lower TSH was an independent risk factor for vertebral fractures [3]. Conversely, removal of the TSHR in the global Tshr−/− mouse leads to high bone turnover and osteoporosis [1,2]. Reddi and Sullivan (1980) experimented the influence of hypophysectomy (hypox) in male rats and administration of GH and TSH on matrix-induced endochondral bone differentiation [5]. In support of this, accumulating evidences showed for the presence of TSHR in rat osteosarcoma cell line [5], human osteoblasts [5] and osteoblast-like SaoS-2 cells [6,7,8,9,10]. TSH is also found to generate T3 through increased expression of deiodinase in human osteoblast like SaOS-2 cells [5]. It promotes osteoblast development in U2OS cells, which is mediated in part by activator signals via β-arrestin-1 [11]. TSH was found to inhibit osteoclast formation and survival through TSHR expressed in these cells. Thus, TSH has definite regulatory effects on bone cells [1,9]. Clinical experiment by Mazziott et al. [4] found that a single subcutaneous injection of recombinant human TSH to postmenopausal osteoporotic women drastically lowers serum C-telopeptide, a marker of bone resorption and also facilitates bone formation.

The primary bone-forming cells, osteoblasts, produce a distinct combination of extracellular matrix proteins. Runx2 regulates the OCN, collagen, and ALP. Studies have revealed that Runx2 involve cells commit to osteogenesis mediating through their target genes, such as ALP, Collagen and OCN. Therefore, Runx2 has a crucial role in osteoblast differentiation [12]. Various hormones like PTH, Estradiol, Growth hormone, Glucocorticoids and growth factors regulate Runx2 activity [13,14,15,16,17,18,19]. Thyroid hormone controls bone matrix synthesis, cell differentiation and degradation of bone matrix [20]. TSH’s downstream signalling pathway is primarily mediated by cAMP, PKA, and CREB. This cAMP-PKA-CREB pathway’s metabolic function in bone has been studied both in vivo and in vitro [21,22]. Due to decreased expression of SP1 and ETS1 during osteoblast differentiation, ETS-like factor ELK1 becomes the primary regulator of Runx2 transcription via the Y-repeat. Increased expression and phosphorylation of ELK1 may correlate with improved Runx2 expression following osteogenic differentiation. ELK1 may transmit signals from the MAPK cascade to induce Runx2 gene expression [23].

In vitro studies demonstrate that TSH regulates rat bone marrow stem cell proliferation, differentiation and apoptosis by direct and indirect mechanisms. In a recent study, Baliram et al. [24] showed that mouse bone marrow (BM) cells produce a novel TSH splice variant (TSH-β). It is produced by macrophages within the bone and BM microenvironment and is capable of signalling to, and enhancing the formation of, local osteoblasts through paracrine communication. Studies conducted in vivo and in vitro have shown effects of TSH on bone. The present study investigated the role of human TSH in bone-forming osteoblastic transcriptional factor Runx2 and its functional genes such as ALP, collagen, and OCN, and matrix mineralization in human osteoblasts such as SaOS-2 and HOS cells in vitro. In addition, a molecular docking analysis was also performed in order to check the binding interaction of Runx2 with CREB and ELK1.

## 2. Results

### 2.1. TSH Induces Runx2 mRNA and Protein Levels in HOS and SaOS2 Cells

**Figure 1:**
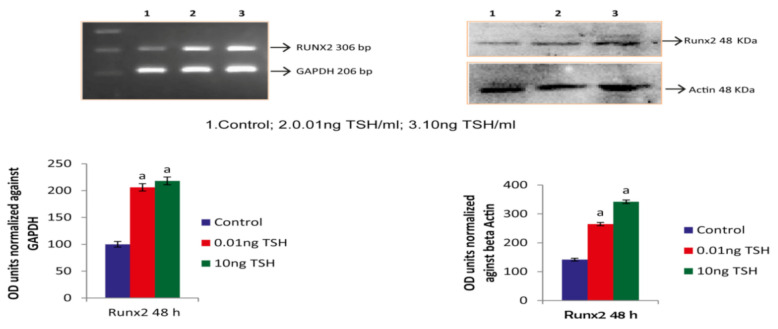
TSH influences Runx2 expression in HOS cells. Each bar diagram shows mean ± SEM of three experiments; ‘a’ represents compared with control at *p* < 0.05.

**Figure 2:**
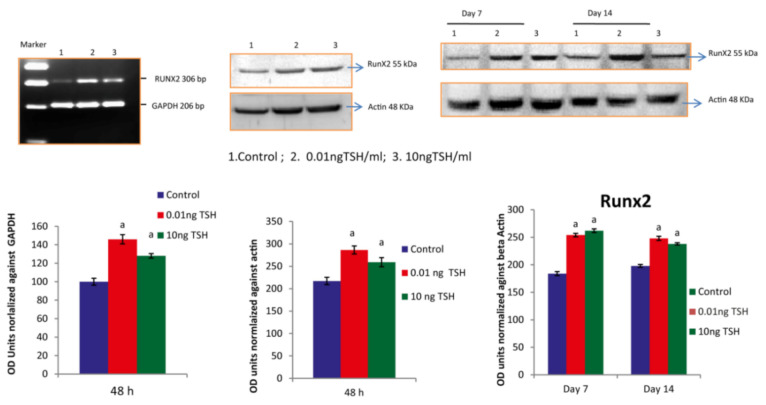
TSH influences Runx2 expression in SaOS-2 cells. Each bar diagram shows mean ± SEM of three experiments; ‘a’ represents compared with control at *p* < 0.05.

Runx2 promotes osteoblastic differentiation through various osteobalstic genes. In the current research, we treated osteoblast like cells of human such as HOS and SaOs-2 with TSH at the concentrations of 0.01 and 10ng/mL at 48 h. After, we isolated total RNA, converted cDNA for assessment of gene expression analysis by RT-PCR to amplify the targeted gene GAPDH and Runx 2. Further, in order to find the levels of role of TSH on the expression of Runx2, we performed western blotting analysis. TSH at a dose of 0.01 ng/mL and 10 ng/mL showed one fold increase in Runx2 mRNA and nearly two fold increase in Runx2 protein level in HOS cells. However, in SaOS-2 cells only fifty percentage increase in Runx2 mRNA and protein level at a dose of 0.01 ng/mL and 10 ng/mL (Figure 1 and Figure 2).

TSH was treated to SaOS-2 cells with various doses for 14 days. Total protein from treated and untreated cells was isolated and used for the analysis of protein expression of day 7 and 14. TSH at the doses of 0.01 and 10 ng/mL, upregulated the Runx2 protein expression when compared to control.

### 2.2. TSH Stimulates IGF-IR in SaOS-2 Cells

**Figure 3:**
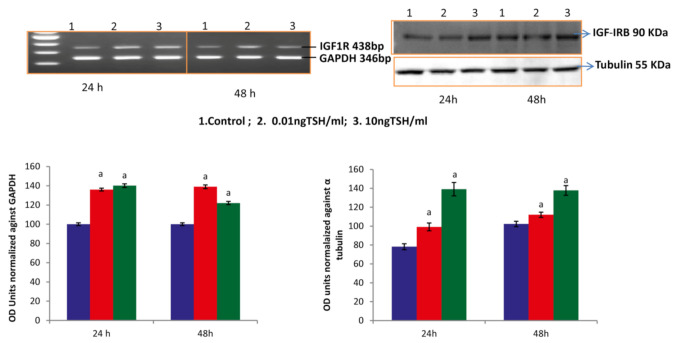
TSH influences expression of IGF-IRB in SaOS-2 cells. Each bar diagram shows mean ± SEM of three experiments; ‘a’ represents compared with control at *p* < 0.05.

Biological action of Runx2 is also facilitated via IGFS. IGFs are mediated through IGF-IR. TSH has been shown to upregulate IGF-1 and IGF-II mRNA and protein expression [9]. In our study, TSH significantly elevated the protein and mRNA expression of IGF-IR at the 0.01 and 10 ng/mL doses on 24 & 48 h durations in SaOS-2 cells. In the present research, the increased expression of IGF-IRB may be due to increase in IGFs by TSH. TSH stimulated IGFs might have acted through IGF-IR in osteoblasts and involved in the expression of Runx2 in these cells (Figure 3).

### 2.3. TSH Promotes mRNA Expression of Differentiation Markers (ALP, COLLAGEN and OCN) in SaOS-2 Cells

TSH was used to see if differentiation marker genes are controlled by TSH. Cells were treated for 14 days (10nM Dexamethasone+50 µg/mL ascorbic acid). Then, the mRNA expression of osteocalcin, ALP and collagen was measured at day 1, day 7, and day 14 in cells that had been treated for 14 days.

**Figure 4:**
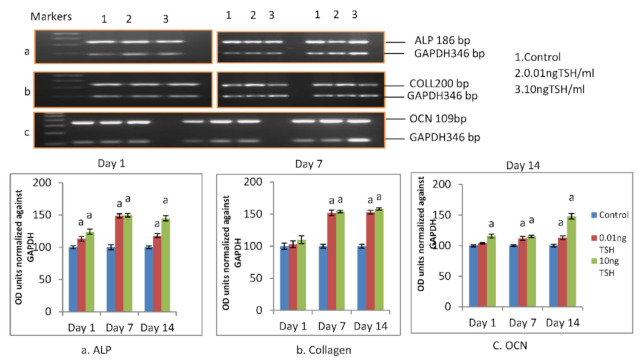
Effect of TSH on Osteocalcin, collagen and ALP mRNA expression in SaOS-2 cells. Each bar diagram shows mean ± SEM of three experiments; ‘a’ represents compared with control at *p* < 0.05.

From day 1 to day 14, the results exhibited a considerable rise in the mRNA expression of osteocalcin, collagen, and AL. At dosages of 0.01 ng/mL and 10 ng/mL TSH, the increase in ALP mRNA was determined to be 48.2% and 49.2% on day 7, respectively, and 20% and 45%on day 14 (Figure 4a).

Compared to control, collagen mRNA was considerably higher in TSH-treated cells. In TSH-treated cells, the increase in collagen mRNA level was 52% and 54% on day 7 and 53% and 58% on day 14, respectively (Figure 4b). The increase in osteocalcin levels following TSH-treatment was dosage dependent. After treatment with 0.01 and 10 ng/mL, the percentage rise in osteocalcin levels (*p* < 0.05) was 12% and 15% on day 7 and 15 percent and 52 percent on day 14, respectively (Figure 4c).

### 2.4. Impact of TSH on Mineralization of Matric in SaOS-2 Cells

**Figure 5:**
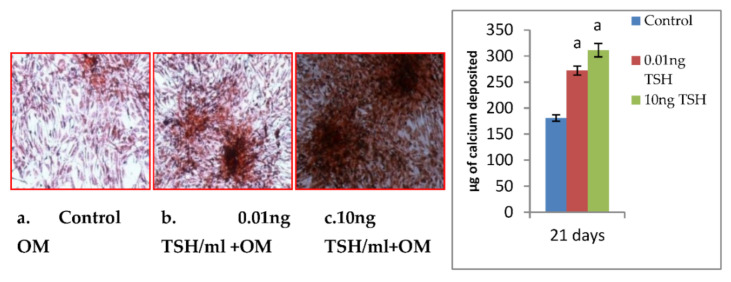
Effect of TSH on mineralization in SaOS-2 cells (Phase contrast Microscopy X 10). Each bar diagram shows mean ± SEM of three experiments; ‘a’ represents compared with control at *p* < 0.05.

The development of bone nodules and the mineralization of extracellular matrix are osteoblastic phenotypic markers that characterize the finishing phases of differentiation of osteoblasts. In order to determine the differentiation end point of SaOS-2 cell cultures, we measured bone nodule development and concentration of calcium. In the mineralization studies, cells were treated with TSH at a dose of 0.01 and 10 ng/mL in SaOS-2 cells. Results of this study showed that compared to the control, TSH increased the production of bone nodules (Figure 5). Depending on the state of matrix maturation and mineral deposition, red patches of mineralized nodules. Alizarin S red staining was used to quantify the amount of calcium accumulated in the extracellular matrix. When compared to control wells, TSH treatment raised the amounts of calcium in the matrix.

### 2.5. Runx2 Homology Modeling

**Figure 6:**
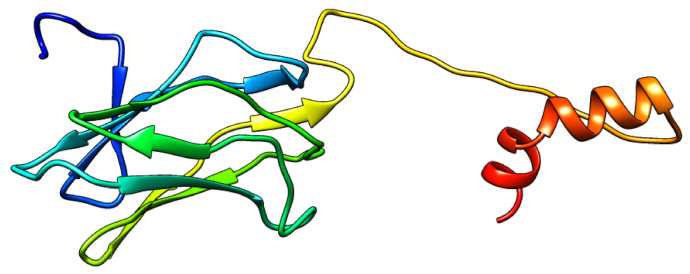
The three-dimensional structure of Runx2 using the SWISS-MODEL.

Homology modelling is a typical method for structure prediction that aids in the study of how protein structure and function interact. Proteins were modelled using the SWISS-MODEL service, which was based on protein structural homology. The SWISS-MODEL server was used to model RUNX-2’s three-dimensional structure. The protein’s GMQE value and QMEAN score indicated that the modelled structure is trustworthy and of high quality. PROCHECK validation also showed that predicted model was as good model. The structure of modeled structure is shown in Figure 6.

### 2.6. Protein-Protein Interactions of RUNX-2 with CREB and ELK

**Figure 7:**
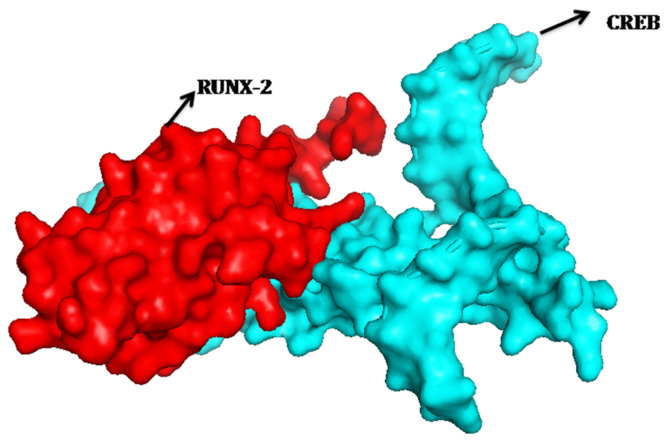
Protein–protein interaction of Runx2 with CREB.

**Figure 8:**
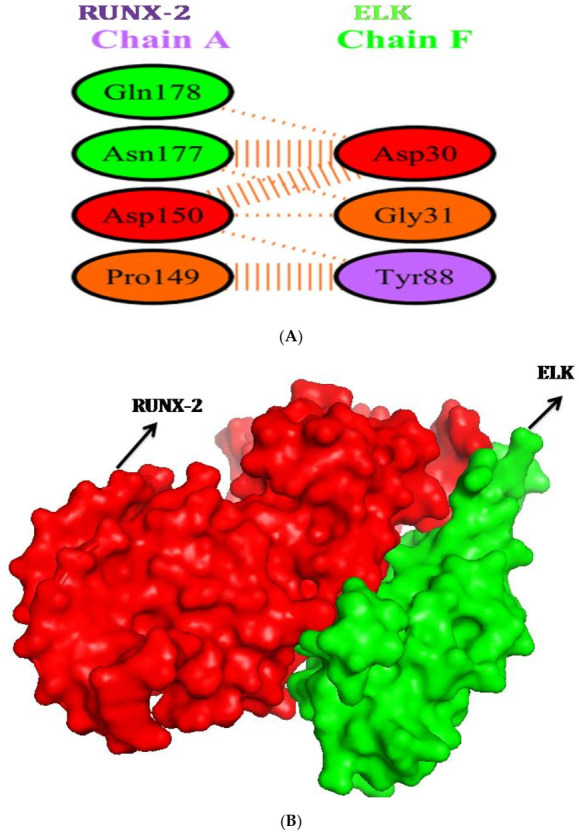
(**A**,**B**): Protein–protein interaction of Runx2 with ELK1.

The creation of protein-protein complexes is required for many biological functions of proteins. As a result, we investigated RUNX-2’s protein-protein interactions with CREB and ELK. The RUNX-2 binding site cleft has been identified as a location for protein interaction, with data from CREB and ELK indicating that interactions occur near the binding site region. When looking at the interactions, RUNX-2 and CREB have a good relationship and create a protein-protein complex (Figure 7). The amino acids GLN-178, ASN-177, ASP-150, and PRO-149 were implicated in the interactions of the ELK protein’s F chain. ELK protein amino acids such as ASP 30, GLY-31, and THR-88 create non-bonded interactions with RUNX2 protein. The interaction figure between RUNX-2 and ELK was shown in Figure 8A,B. Likewise the amino acids THR-324, LEU325, TYR-307, ASN-313, VAL-317, ARG-314, LEU-311 of CREB form the interaction with RUNX-2 protein.

## 3. Discussion

The regulation of osteoblast differentiation and mineralization is essential to maintain bone mass. Various hormones regulate the differentiation and mineralization of bone forming osteoblasts. In this study, upregulation of mRNA and protein in response to TSH clearly demonstrates that TSH facilitates osteoblasts’ functional activity. Runx2 has also been found to be highly expressed in BHP-cultured papillary carcinoma cells and surgically excised human thyroid cancer tissues [25]. TSH also induces Runx2 mRNA expression in rat thyroid cells (FRTL-5), which in turn stimulates osteocalcin type-I collagen and alkaline phosphatase expression in FRTL-5 cells [25]. These findings clearly indicate that TSH acts in a similar fashion in thyrocytes as well as in osteoblasts. If so, it is essential to know the mechanism of TSH action in this regard. According to Morimura et al. [9], TSH activates Protein kinase A (PKA) in SaOS2 cells. It’s reasonable to presume that TSH would have enhanced Runx2 expression in HOS and SaOS-2 cells by activating PKA signalling pathways.

Activity of a transcriptional factor can be regulated by post translational modification such as phosphorylation, acetylation etc. The Runx2 protein has many phosphorylation sites, and phosphorylation at different locations has stimulatory or inhibitory functions [26,27,28] showed that Runx2 is phosphorylated at the Ser247 through PKC-δ signalling associated with increase in transcriptional activity of Runx2. In embryonic stem cell cultures, TSH has been demonstrated to activate PKC than PKA, and to transduce a non-traditional, non-cAMP-dependent signal in preadipocytes and thyrocytes [29]. Boutin et al. (2014), [11] have shown that TSH stimulates β-arrestin-1, which leads to the activation of intracellular signalling molecules such as ERK, P38 MAPK, and AKT in human osteoblastic U2OS-cells and incuses Osteopontin, ALP and RANKL genes. TSH may have triggered active forms of ERK, P38 MAPK, and AKT, which in turn stimulates Runx2 expression in HOS and SaOS-2 cells by activation of any of these signalling systems.

Apart from the direct action of TSH on Runx2 expression, there is a possibility for the indirect effect of TSH through IGFs. Previous study out laboratory has shown that TSH increases the expression of IGF-I and II both at mRNA and protein levels [10]. Findings of the present study clearly shows that TSH stimulated IGFs which in turn increased the expression of IGF-IR in human osteoblastic cells. IGF IR also favors Runx2 elevation through ERK and p38 MAPK. IGFS is reported to increase the mRNA expression of Runx2 in MC3T3-E1 and C2C12 cells [30]. Probably, TSH could have mediated their stimulatory effects on Runx2 expression through IGFs.

ALP gene is abundantly expressed in the extracellular matrix, is rendered competent for mineralization. In the present study, TSH significantly increased ALP mRNA expression and its activity, mRNA expression of collagen and osteocalcin. TSH has been reported to increase ALP activity [10], matrix mineralization in rat primary calvariae-derived osteoblastic cells, mouse pre-osteoblastic cells and rat osteosarcoma-derived osteoblastic [2], as well as in mouse generated embryonic stem cells [31]. TSH also increases ALP expression in U2OS cells via β-arrestin-1-mediated activation signals [11]. TSH causes a rise in ALP activity and matrix mineralization in these cells, indicating that it has a beneficial influence on osteoblast development. The regulatory region of collagen is found to contain AP-1, NF-1, SP-1, C/EBP, NF kB, Smad and Runx2 binding sites. Many transcription factors interact with these upstream regulatory elements and control the basal transcription [32]. In a recent study, TSH is reported to trigger a dramatic increase (200 fold) in the expression of type-1 collagen in murine embryonic stem cells in addition to the increase in ALP and osteocalcin [30]. Osteocalcin is a late marker of osteoblastic maturation that is linked to the development of osteoblasts [33]. Osteocalcin is formed in the osteoblast and then deposited in bone matrix. Osteoblast differentiation is a critical step in the development, repair, and maintenance of skeletal tissue. The expression of cell-specific transcription factors is required for osteoblast development [34]. Runx2 regulates the expression of all key osteoblast-specific genes such as type-I collagen, ALP, osteocalcin, BSP, and OPN by binding to the osteoblast specific cis acting element (OSE) present in the promoter region [35]. TSH-induced Runx2 protein was found to be responsible for increased osteocalcin, ALP and collagen mRNA levels in the current study. The ability of an osteogenic tissue to manifest its phenotypic expression is its ultimate phenotypic expression to create an extracellular matrix that can undergo controlled mineralization. When compared to untreated cells, TSH increased the development of mineralized nodules after 21 days of SaOS-2 cell growth. The calcium concentration in the matrix was measured using the Alizarin S Red test to determine the mineralization. TSH stimulates mineralization of matrix released by rat and mouse osteoblastic cells, according to previous research [2,30]. Increased mineralization was accompanied by a rise in osteocalcin levels, as measured by Alizarin red staining. Matrix mineralization is aided by osteocalcin. It has been found to perform a favorable impact in hydroxyapatite crystal nucleation regulation. Osteocalcin governs bone crystal formation by binding to hydroxyapatite crystals, the primary mineral component of bone [36]. The present study also revealed coordinated up regulation of osteocalcin and nodule formation after TSH treatment in SaOS2 cells in vitro. TSH is known to be activated via cAMP or Protein kinase-C pathway. Present in silico study was confirmed that CREB and ELK1 interaction with Runx2. Hence, TSH downstream molecules interaction with Runx2 may induce osteoblastic specific functional genes. To the best of our knowledge this is the first study showing Runx2 interaction with CREB and ELK1. Future studies to be warranted to elucidate in experimental animal models. Thus, the anabolic effects of TSH on bone formation are affected by increasing osteoblastic Runx2 mediated differentiation and matrix mineralization.

## 4. Materials and Methods

### 4.1. Culturing and Maitenance of Cells

The National Centre for Cell Sciences (NCCS), India, provided SaOS-2 and HOS cells. The cells were cultivated until confluence in DMEM with 10% FBS. The cells were then employed for subculture or treatment after being separated with trypsin EDTA.

### 4.2. Chemicals

All chemical and reagents used in the current study were procured from Sigma, St. Louis, MO, USA, and GIBCO-BRL, New York, NY, USA.

### 4.3. Gene Expression Study

At the appropriate time points, total RNA was isolated in HOS and SaOS-2 cells and quantified spectrophotometrically and reverse transcriptase enzyme was used to convert 1.5 µg of total RNA to complementary DNA (Qiagen, Hilden, Germany). Gene specific primers of Collagen, Runx2, OCN and ALP were used in PCR. Table 1 lists primers employed in our study. PCR steps consisting of denaturation at 94 °C for 1.5 min, annealing at 55 °C for 1.5 min, and extension at 72 °C for 1.5 min. Expression of targeted gene was quantified using quantity-One-Software (Bio-Rad, Hercules, CA, USA).

### 4.4. Protein Expression Study

Cell lysate were prepared as per the method of Selvamurugan et al. [12]. Briefly, 50 µg of proteins used to separate on 12% SDS-PAGE and transferred on to PVDF (Bio-Rad) membrane. After blocking the membrane with 5% non-fat dried milk, it was incubated with IGF-IR primary antibody at overnight at 4 °C. The membrane was then exposed to HRP-conjugated secondary antibody. Enhanced chemiluminescence detection kit (Sigma, St. Louis, MO, USA) was used to visualize the signals.

### 4.5. Assessment of Mineralization of Matrix

In our study, matrix mineralization was done as per the method of Johnson et al. [37]. The alizarin red stained cells were treated with 100 mM cetylpyridinium chloride for 1 h to solubilize and release calcium-bound alizarin red into solution in order to measure matrix mineralization the absorbance was measured at 570 nm.

### 4.6. Molecular Docking Study

#### 4.6.1. Retrieval of Protein Structures

The interaction mechanism of RUNX-2 with CREB and ELK was investigated in this study for determination of interaction among these proteins. To facilitate this interaction, the two proteins structures were downloaded from the Protein Data Bank. The 3D structures of proteins were retrieved as follows CREB (Pdb id: 5ZK1), ELK (Pdb id: 1DUX) and since RUNX-2 three dimensional structure was not available in the Protein Data Bank, we modeled using homology modeling approach.

#### 4.6.2. Homology Modeling of RUNX-2

RUNX2 Human’s amino acid sequence was obtained from the Uniprot database (ID: Q13950) [38,39,40]. BLASTP search against the Brookhaven Protein Data Bank with default parameters was used to locate relevant templates for homology modelling. The Runx1 and Ets 1 crystal structure bound to the TCR-α promoter with 72.84 percent identity was chosen as a template for creating three-dimensional structure based on maximal identity, high score, and low e-value. The RUNX-2 model was created using the SWISS-MODEL server [34]. The SWISS-MODEL web server calculates the QMEAN scoring function automatically to approximate the local and global model quality based on the geometry, interactions, and solvent capacity of the protein model. The PROCHECK tool was used to validate the stereochemical characteristics of the modelled structure.

#### 4.6.3. Protein–Protein Docking

The RUNX-2 was docked with CREB and ELK using a geometry-based molecular docking technique called Patch Dock (http://bioinfo3d.cs.tau.ac.il/PatchDock, accessed on 1 December 2021) [41,42,43].

#### 4.6.4. Visualization of Protein–Protein Interactions

Pymol was used to study, the residual interactions between RUNX-2 with CREB and ELK. The color intensity for interactions was clearly visible here, and the findings were exported. Pdbsum was used to identify the types of interaction occurs between RUNX-2 with CREB and ELK proteins [44,45,46,47].

### 4.7. Data Analysis

Using computer-based software, SPSS the data were statistically analysed using One-way- ANOVA followed by the Student-Newman-Keul (SNK) test for multiple comparisons. *p* value <0.05 was considered to have a pronounced difference between the groups.

## 5. Conclusions

To conclude, the TSH’s anabolic effects on the formation of bone are facilitated by enhancing osteoblastic Runx2-mediated differentiation and matrix mineralization. We report for the first time the possible role of TSH on RUNX-2 binding with the CREB and ELK proteins in HOS and SaOS-2 human osteoblastic like cells.

## Figures and Tables

**Table 1:** List of primers was used in this study.

Gene	Forward Primer	Reverse Primer	Size (bp)	Accession No.
ALP	ACCTCGTTGACACCTGGAAG	CCACCATCTCGGAGAGTGAC	189	NM_000478
Collagen	GGCCCAGAAGAACTGGTAC	CGCTGTTCTTGCAGTGGTAG	200	NM_000088
Runx2	ACCTCGTTGACACCTGGAAG	TCTCGGTGGCTGGTAGTGA	309	NM_001015051
IGF1R	TGGGGAATGGAGTGCTGTAT	ACGTTTGGCCTCCCTGAACG	438	NM_001291858.2
OCN	GAATGGTCACTGGGCTGTTT	CTGGCAGCTTTGCACAATTA	109	NM_199173.4
GAPDH	GGAGTCAACGGATTTGGT	ATCAATGGAAATCCCATCAC	206	NM_002046
GAPDH	GCTCTCCAGAACATCATCCCT	CGTTGTCATACCAGGAAATGAGCT	346	NM_001289745

## Data Availability

The data presented in this study are available in this article.

## Sample Availability

Samples of the compounds are not available from the authors.

## References

[1] Abe E., Marians R.C., Yu W., Wu X.B., Ando T., Li Y., Iqbal J., Eldeiry L., Rajendren G., Blair H.C. (2003). TSH is a negative regulator of skeletal remodeling. Cell.

[2] Sampath T.K., Simic P., Sendak R., Draca N., Bowe A.E., O’Brien S., Schiavi S.C., McPherson J.M., Vukicevic S. (2007). Thyroid-stimulating hormone restores bone volume, microarchitecture, and strength in aged ovariectomized rats. J. Bone Miner. Res..

[3] Sun L., Vukicevic S., Baliram R., Yang G., Sendak R., McPherson J., Zhu L.L., Iqbal J., Latif R., Natrajan A. (2008). Intermittent recombinant TSH injections prevent ovariectomy-induced bone loss. Proc. Natl. Acad. Sci. USA.

[4] Mazziotti G., Sorvillo F., Piscopo M., Cioffi M., Pilla P., Biondi B., Iorio S., Giustina A., Amato G., Carella C. (2005). Recombinant human TSH modulates in vivo C-telopeptides of type-1 collagen and bone alkaline phosphatase, but not osteoprotegerin production in postmenopausal women monitored for differentiated thyroid carcinoma. J. Bone Miner. Res..

[5] Reddi A.H., Sullivan N.E. (1980). Matrix-induced endochondral bone differentiation: Influence of hypophysectomy, growth hormone, and thyroid-stimulating hormone. Endocrinology.

[6] Inoue M., Tawata M., Yokomori N., Endo T., Onaya T. (1998). Expression of thyrotropin receptor on clonal osteoblast-like rat osteosarcoma cells. Thyroid.

[7] Williams G.R. (2009). Does serum TSH level have thyroid hormone independent effects on bone turnover?. Nat. Clin. Pract. Endocrinol. Metab..

[8] Tsai J.A., Janson A., Bucht E., Kindmark H., Marcus C., Stark A., Zemack H.R., Torring O. (2004). Weak evidence of thyrotropin receptors in primary cultures of human osteoblast-like cells. Calcif. Tissue Int..

[9] Morimura T., Tsunekawa K., Kasahara T., Seki K., Ogiwara T., Mori M., Murakami M. (2005). Expression of type 2 iodothyronine deiodinase in human osteoblast is stimulated by thyrotropin. Endocrinology.

[10] Ramajayam G., Vignesh R.C., Karthikeyan S., Kumar K.S., Karthikeyan G.D., Veni S., Sridhar M., Arunakaran J., Aruldhas M.M., Srinivasan N. (2012). Regulation of insulin-like growth factors and their binding proteins by thyroid stimulating hormone in human osteoblast-like (SaOS2) cells. Mol. Cell. Biochem..

[11] Boutin A., Eliseeva E., Gershengorn M.C., Neumann S. (2014). β-Arrestin-1 mediates thyrotropin-enhanced osteoblast differentiation. FASEB J..

[12] Komori T. (2010). Regulation of osteoblast differentiation by Runx2. Adv. Exp. Med. Biol..

[13] Selvamurugan N., Pulumati M.R., Tyson D.R., Partridge N.C. (2000). Parathyroid hormone regulation of the rat collagenase-3 promoter by protein kinase A-dependent transactivation of core binding factor alpha1. J. Biol. Chem..

[14] McCarthy T.L., Chang W.Z., Liu Y., Centrella M. (2003). Runx2 integrates estrogen activity in osteoblasts. J. Biol. Chem..

[15] Tian Y., Xu Y., Fu Q., He M. (2011). Parathyroid hormone regulates osteoblast differentiation in a Wnt/β-catenin-dependent manner. Mol. Cell. Biochem..

[16] Vanderschueren D., Vandenput L., Boonen S., Lindberg M.K., Bouillon R., Ohlsson C. (2004). Androgens and bone. Endocr. Rev..

[17] Hubina E., Lakatos P., Kovács L., Szabolcs I., Rácz K., Tóth M., Szucs N., Góth M.I. (2004). Effects of 24 months of growth hormone (GH) treatment on serum carboxylated and undercarboxylated osteocalcin levels in GH-deficient adults. Calcif. Tissue Int..

[18] Giustina A., Mazziotti G., Canalis E. (2008). Growth hormone, insulin-like growth factors, and the skeleton. Endocr. Rev..

[19] Moutsatsou P., Kassi E., Papavassiliou A.G. (2012). Glucocorticoid receptor signaling in bone cells. Trends Mol. Med..

[20] Varga F., Rumpler M., Zoehrer R., Turecek C., Spitzer S., Thaler R., Paschalis E.P., Klaushofer K. (2010). T3 affects expression of collagen I and collagen cross-linking in bone cell cultures. Biochem. Biophys. Res. Commun..

[21] Tintut Y., Patel J., Parhami F., Demer L.L. (2000). Tumor necrosis factor-alpha promotes in vitro calcification of vascular cells via the cAMP pathway. Circulation.

[22] Huang W.C., Xie Z., Konaka H., Sodek J., Zhau H.E., Chung L.W. (2005). Human osteocalcin and bone sialoprotein mediating osteomimicry of prostate cancer cells: Role of cAMP-dependent protein kinase A signaling pathway. Cancer Res..

[23] Zhang Y., Hassan M.Q., Xie R.L., Hawse J.R., Spelsberg T.C., Montecino M., Stein J.L., Lian J.B., van Wijnen A.J., Stein G.S. (2009). Co-stimulation of the bone-related Runx2 P1 promoter in mesenchymal cells by SP1 and ETS transcription factors at polymorphic purine-rich DNA sequences (Y-repeats). J. Biol. Chem..

[24] Baliram R., Sun L., Cao J., Li J., Latif R., Huber A.K., Yuen T., Blair H.C., Zaidi M., Davies T.F. (2012). Hyperthyroid-associated osteoporosis is exacerbated by the loss of TSH signaling. J. Clin. Investig..

[25] Endo T., Ohta K., Kobayashi T. (2008). Expression and function of Cbfa-1/Runx2 in thyroid papillary carcinoma cells. J. Clin. Endocrinol. Metab..

[26] Franceschi R.T., Xiao G., Jiang D., Gopalakrishnan R., Yang S., Reith E. (2003). Multiple signaling pathways converge on the Cbfa1/Runx2 transcription factor to regulate osteoblast differentiation. Connect. Tissue Res..

[27] Kim B.G., Kim H.J., Park H.J., Kim Y.J., Yoon W.J., Lee S.J., Ryoo H.M., Cho J.Y. (2006). Runx2 phosphorylation induced by fibroblast growth factor-2/protein kinase C pathways. Proteomics.

[28] Rivas M., Santisteban P. (2003). TSH-activated signaling pathways in thyroid tumorigenesis. Mol. Cell. Endocrinol..

[29] Baliram R., Latif R., Berkowitz J., Frid S., Colaianni G., Sun L., Zaidi M., Davies T.F. (2011). Thyroid-stimulating hormone induces a Wnt-dependent, feed-forward loop for osteoblastogenesis in embryonic stem cell cultures. Proc. Natl. Acad. Sci. USA.

[30] Ihn H., Ohnishi K., Tamaki T., LeRoy E.C., Trojanowska M. (1996). Transcriptional regulation of the human alpha2(I) collagen gene. Combined action of upstream stimulatory and inhibitory cis-acting elements. J. Biol. Chem..

[31] Pei Y., Meng X.W., Zhou X.Y., Xing X.P., Xia W.B. (2003). Expression of core binding factor alpha1 up-regulated by IGF-I, GM-CSF, and EGF through MAPK pathway in MC3T3-E1 and C2C12 cells. Acta Pharmacol. Sin..

[32] Aubin J.E., Liu F., Malaval L., Gupta A.K. (1995). Osteoblast and chondroblast differentiation. Bone.

[33] Lian J.B., Stein G.S., Stein J.L., van Wijnen A.J. (1998). Osteocalcin gene promoter: Unlocking the secrets for regulation of osteoblast growth and differentiation. J. Cell. Biochem..

[34] Ducy P., Starbuck M., Priemel M., Shen J., Pinero G., Geoffroy V., Amling M., Karsenty G. (1999). A Cbfa1-dependent genetic pathway controls bone formation beyond embryonic development. Genes Dev..

[35] Boskey A.L. (1998). Biomineralization: Conflicts, challenges, and opportunities. J. Cell. Biochem..

[36] UniProt Consortium (2019). UniProt: A worldwide hub of protein knowledge. Nucleic Acids Res..

[37] Johnson K., Hashimoto S., Lotz M., Pritzker K., Goding J., Terkeltaub R. (2001). Up-regulated expression of the phosphodiesterase nucleotide pyrophosphatase family member PC-1 is a marker and pathogenic factor for knee meniscal cartilage matrix calcification. Arthritis Rheum..

[38] Waterhouse A., Bertoni M., Bienert S., Studer G., Tauriello G., Gumienny R., Heer F.T., de Beer T.A.P., Rempfer C., Bordoli L. (2018). SWISS-MODEL: Homology modelling of protein structures and complexes. Nucleic Acids Res..

[39] Schneidman-Duhovny D., Inbar Y., Nussinov R., Wolfson H.J. (2005). PatchDock and SymmDock: Servers for rigid and symmetric docking. Nucleic Acids Res..

[40] Pettersen E.F., Goddard T.D., Huang C.C., Couch G.S., Greenblatt D.M., Meng E.C., Ferrin T.E. (2004). UCSF Chimera—A visualization system for exploratory research and analysis. J. Comput. Chem..

[41] Maruthamuthu M.K., Ganesh I., Ravikumar S., Hong S.H. (2015). Evaluation of zraP gene expression characteristics and construction of a lead (Pb) sensing and removal system in a recombinant Escherichia coli. Biotechnol. Lett..

[42] Somasundaram S., Maruthamuthu M.K., Ganesh I., Eom G.T., Hong S.H. (2017). Enchancement of Gamma-Aminobutyric Acid Production by Co-Localization of Neurospora crassa OR74A Glutamate Decarboxylase with Escherichia coli GABA Transporter Via Synthetic Scaffold Complex. J. Microbiol. Biotechnol..

[43] Kannan M.M., Vanitha J., Jiang S., Ramachandran S. (2013). Impact of colchicine treatment on sorghum bicolor BT × 623. Mol. Plant Breed..

[44] Maruthamuthu M.K., Hong J., Arulsamy K., Somasundaram S., Hong S., Choe W.S., Yoo I.K. (2018). Development of bisphenol A-removing recombinant Escherichia coli by monomeric and dimeric surface display of bisphenol A-binding peptide. Bioprocess Biosyst. Eng..

[45] Davidson J.L., Wang J., Maruthamuthu M.K., Dextre A., Pascual-Garrigos A., Mohan S., Putikam S.V.S., Osman F.O.I., McChesney D., Seville J. (2021). A paper-based colorimetric molecular test for SARS-CoV-2 in saliva. Biosens. Bioelectron. X.

[46] Maruthamuthu M.K., Nadarajan S.P., Ganesh I., Ravikumar S., Yun H., Yoo I.K., Hong S.H. (2015). Construction of a high efficiency copper adsorption bacterial system via peptide display and its application on copper dye polluted wastewater. Bioprocess Biosyst. Eng..

[47] Maruthamuthu M.K., Selvamani V., Nadarajan S.P., Yun H., Oh Y.K., Eom G.T., Hong S.H. (2018). Manganese and cobalt recovery by surface display of metal binding peptide on various loops of OmpC in Escherichia coli. J. Ind. Microbiol. Biotechnol..

